# Integration of Genetic and Imaging Data to Detect QTL for Root Traits in Interspecific Soybean Populations

**DOI:** 10.3390/ijms26031152

**Published:** 2025-01-28

**Authors:** Mohammad Shafiqul Islam, Jeong-Dong Lee, Qijian Song, Hyun Jo, Yoonha Kim

**Affiliations:** 1 Department of Applied Biosciences, Kyungpook National University, Daegu 41566, Republic of Korea; shafik.hort@gmail.com (M.S.I.); jdlee@knu.ac.kr (J.-D.L.); 2 Department of Integrative Biology, Kyungpook National University, Daegu 41566, Republic of Korea; 3 Department of Agriculture, Noakhali Science and Technology University, Noakhali 3814, Bangladesh; 4 Upland Field Machinery Research Center, Kyungpook National University, Daegu 41566, Republic of Korea; 5 Soybean Genomics and Improvement Laboratory, USDA-ARS, Beltsville Agricultural Research Center, Beltsville, MD 20705, USA; qijian.song@usda.gov

**Keywords:** soybean, root morphological traits, RILs, QTL, SNP, candidate genes

## Abstract

Wild soybean, which has many desirable traits, such as adaptability to climate change-related stresses, is a valuable resource for expanding the narrow genetic diversity of cultivated soybeans. Plants require roots to adapt to different environments and optimize water and nutrient uptake to support growth and facilitate the storage of metabolites; however, it is challenging and costly to evaluate root traits under field conditions. Previous studies of quantitative trait loci (QTL) have been mainly based on cultivated soybean populations. In this study, an interspecific mapping population from a cross between wild soybean ‘PI483463’ and cultivar ‘Hutcheson’ was used to investigate QTLs associated with root traits using image data. Our results showed that 39 putative QTLs were distributed across 10 chromosomes (chr.). Seventeen of these were clustered in regions on chr. 8, 14, 15, 16, and 17, accounting for 19.92% of the phenotypic variation. We identified five significant QTL clusters influencing root-related traits, such as total root length, surface area, lateral total length, and number of tips, across five chr., with favorable alleles from both wild and cultivated soybeans. Furthermore, we identified eight candidate genes controlling these traits based on functional annotation. These genes were highly expressed in root tissues and directly or indirectly affected soybean root growth, development, and stress responses. Our results provide valuable insights for breeders aiming to optimize soybean root traits and leveraging genetic diversity from wild soybean species to develop varieties with improved root morphological traits, ultimately enhancing overall plant growth, productivity, and resilience.

## 1. Introduction

Roots serve as a fundamental component of plant growth and development. They acquire water and nutrients from the soil, provide mechanical support, facilitate metabolite storage, and anchor the plant within the soil [1,2,3,4]. Crop plants require a strong and expansive root system to adapt to diverse environments and optimize water and nutrition uptake, particularly under input-limited conditions [5,6,7]. However, due to their complexity and adaptability, understanding root systems is challenging, especially when studied in natural and uncontrolled environments [1]. Consequently, research on root traits often lags behind research on above-ground plant traits, and in particular, research on genetic aspects of soybean root morphology in field settings remains relatively limited.

Soybean (*Glycine max* L. Merril) is a vital legume crop and a major global commodity, significantly contributing to food security with diverse uses for humans and animals [8,9]. The growing demand for soybean-based foods and products makes it essential to enhance soybean yield. Studies show that soybean genotypes with early rapid root growth, multiple taproots, and extensive lateral roots demonstrate greater resilience to adverse environmental stresses and the potential for improved yield [10,11,12]. Furthermore, research on various soybean root traits shows genetic variability in key parameters such as total root length (TRL), surface area (SA), root volume, root diameter, lateral root length, link average diameter, root elongation, and fibrous roots [1,3,13,14,15]. The substantial variability observed in soybean root traits indicates the potential to enhance soybean performance through genetic modification targeted at root characteristics.

Cultivated soybean germplasm exhibits narrow genetic diversity, while exotic germplasm, including wild soybeans, serves as a significant genetic resource for expanding the genetic diversity of soybeans, thereby enhancing disease and pest resistance [16,17]. Wild soybean also has drought resistance due to its root characteristics; therefore, interspecific crossing would be beneficial in transferring desirable traits from wild relatives to cultivated varieties [18,19,20]. This study focuses on an interspecific mapping population and identifies wild soybean alleles that could enhance genetic diversity and improve root morphology in cultivated soybeans.

Root morphology plays a crucial role in plant resource uptake, and various root ideotypes serve as potential breeding targets for developing genotypes that are resilient to climate variations [21,22]. The modulation of root morphology represents a complex process influenced by the interaction of genetic and environmental factors [23,24]. However, screening root traits in soybean breeding populations is both challenging and costly, primarily due to the inherent difficulties in accurately quantifying root characteristics under field conditions [1,10]. Quantitative trait loci (QTL) analysis detects specific chromosomal regions that contribute to phenotypic variation in root morphology [15,25]. This approach identifies and characterizes desirable alleles at these QTLs, which can be utilized for marker-assisted selection in breeding programs. To date, researchers have identified several QTLs associated with root morphological traits such as total root length, surface area, root volume, root diameter, primary root length, and link average diameter in soybeans using an inter-specific mapping population [1,3,15,20,26,27,28]. However, studies on interspecific root mapping are limited; therefore, our research aims to investigate interspecific soybean root morphological traits. Furthermore, to reduce the phenotyping of root traits, we used a computer-based image analysis software (WinRHIZO pro version 2019) known for its accuracy in quantifying root morphological characteristics.

In this study, we employed an interspecific mapping population generated by crossing the cultivated soybean variety “Hutcheson” with the wild soybean accession “PI483463”. A linkage map was developed using 3K single-nucleotide polymorphism (SNP) markers. The study aims to identify important QTLs and potential genes associated with significant genomic regions that regulate seedling root morphology in an interspecific soybean mapping population.

## 2. Results

### 2.1. Diversity of Root Traits in the RIL Population

**Figure 1:**
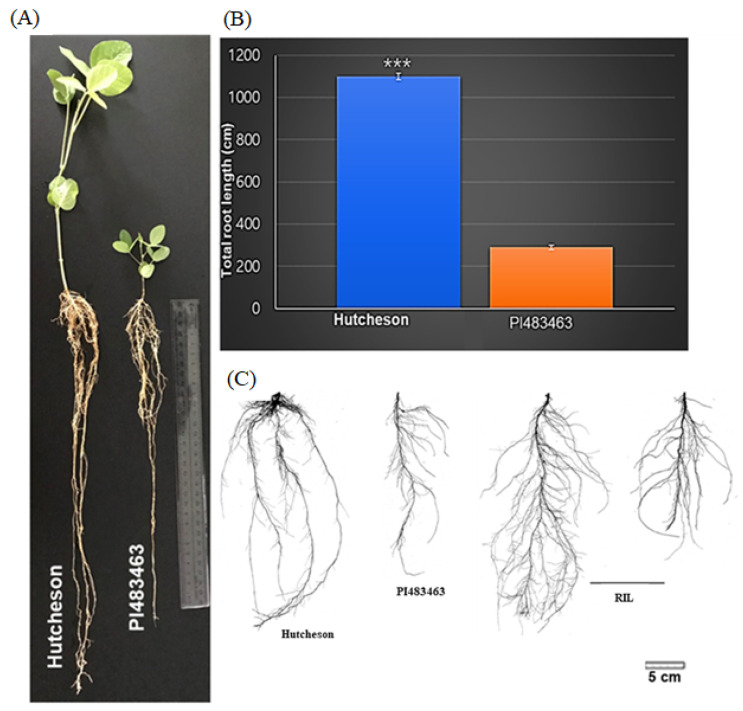
Variation in seedling root morphology of the soybean RIL population. (**A**) Variation in root phenotypes between the cultivated soybean “Hutcheson” and wild soybean “PI483463.” (**B**) Significant variation in TRL between “Hutcheson” and “PI483463.” (**C**) Variation in 2D root image morphology among the parents and RILs. *** *p* < 0.001 (Student’s *t*-test) and the error bar indicates standard error.

The RIL population resulting from a cross between the cultivar “Hutcheson” and wild soybean “PI483463” exhibited diverse root phenotypes (Figure 1) [29]. The TRL of the parental lines “Hutcheson” and “PI483463” showed significant variation (Figure 1B). The RILs demonstrated diverse morphological variations for root length, showing longer or shorter roots (transgressive segregation) compared to their parents (Table 1, Figure 1C).

**Figure 2:**
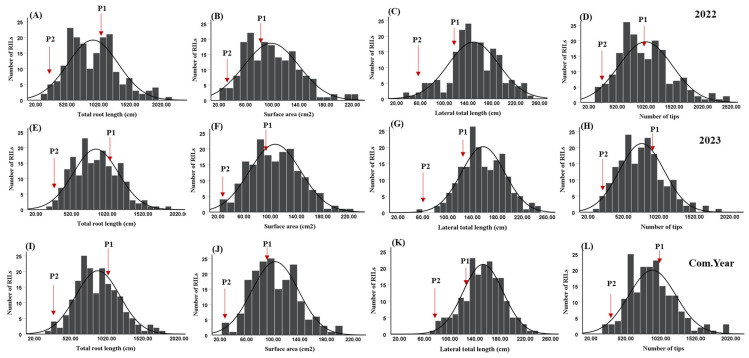
The frequency distribution with the normal curve of four root traits of TRL, SA, LTL, and NT in soybean across different years. (**A**–**D**) TRL, SA, LTL, and NT for the year 2022, respectively; (**E**–**H**) TRL, SA, LTL, and NT for the year 2023, respectively; (**I**–**L**) TRL, SA, LTL, and NT for the combined year, respectively; Arrows indicate the mean values of cultivated parent P1 (“Hutcheson”) and wild parent P2 (“PI483463”); TRL, total root length; SA, surface area; LTL, lateral total length; and NT, number of tips.

The frequencies of the RIL root traits, such as TRL, SA, LTL, and NT, were normally distributed (Figure 2). In 2022, the mean value of TRL among RILs was 934.46 cm with values ranging from 153.65 cm to 2166.03 cm. Other root traits, such as SA, LTL, and NT, exhibited values of 17.02–228.61 cm^2^, 32.68–244.53 cm, and 170.00–2494.00 (number), with corresponding mean values of 98.93 cm^2^, 149.37 cm, and 1019.55, respectively. In the 2023 experiment, the values for TRL, SA, LTL, and NT were uniformly distributed across the population, with values ranging from 246.91 to 1849.63 cm, from 25.42 to 219.17 cm^2^, from 56.30 to 249.17 cm, and from 162.33 to 1841.50 (number), with mean values of 861.87 cm, 107.51 cm^2^, 157.10 cm, and 780.70, respectively. The average TRL, SA, LTL, and NT over the combined years were 898.16 cm, 103.22 cm^2^, 153.23 cm, and 900.12, respectively (Table 1). Based on the results, the skewness and kurtosis values for the measured root traits were less than one in both years, with negative kurtosis observed for TRL, SA, and LTL in 2023 (Table 1).

Statistically significant variations were observed for genotypes and the genotype–environment interaction (*p* < 0.0001) across all root traits (Table 2). The heritability values for TRL, SA, LTL, and NT were 70.8%, 75.7%, 68.2%, and 70.0%, respectively (Table 2). Significant positive correlations (*p* < 0.0001) were observed between any two-root traits in both research years. Among the root traits, TRL exhibited a high positive correlation with SA (r = 0.97, *p* < 0.0001), LTL (r = 0.77, *p* < 0.0001), and NT (r = 0.91, *p* < 0.0001) (Table 3).

### 2.2. QTLs for Root Traits

The genetic linkage maps from the population spanned a total length of 3256.8 cM, with an average distance of 2.74 cM between adjacent markers. The maximum and minimum lengths of individual chr. or linkage groups were 248.3 cM and 123.7 cM, respectively (Table S1). Similarly, chr. 15 had the highest number of markers (82 SNPs), while chr. 12 had the lowest number of markers (36 SNPs) (Table S1, Figure S1).

**Figure 3:**
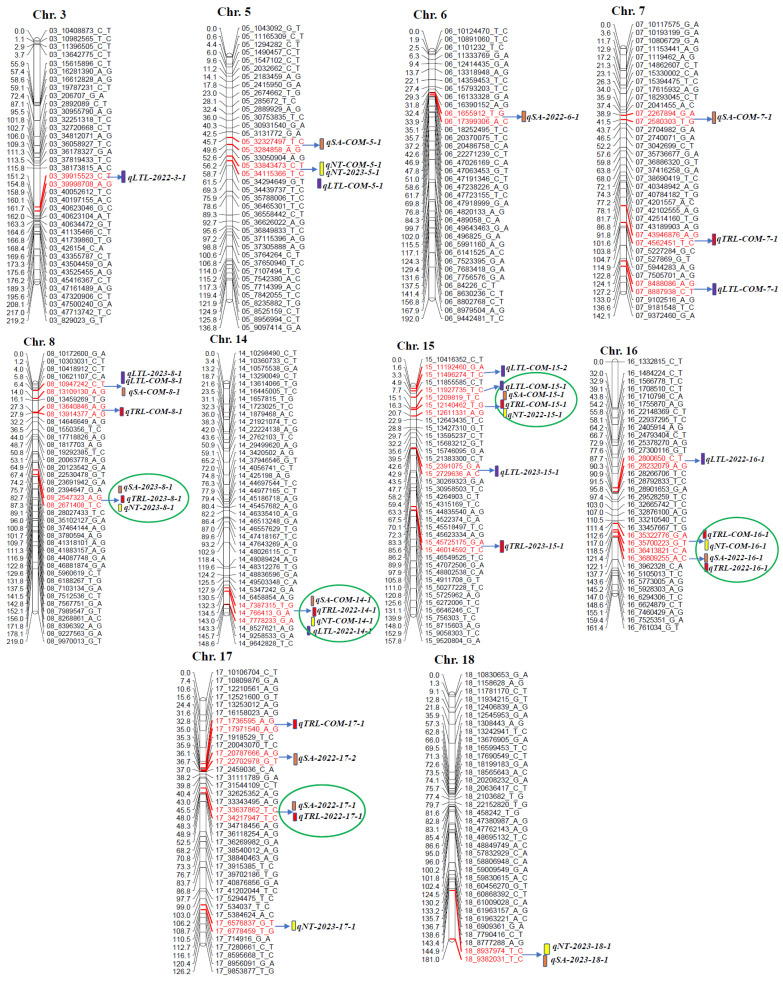
Diagram showing the position of root QTLs on different chromosomes; chr. 3, 5, 6, 7, 8, 14, 15, 16, 17, and 18 in an interspecific soybean population. Within each chromosome, the genetic distance of markers are labeled on the left side, while the name of markers are on right side. Colored bars indicate QTLs. Note: Chr., chromosome; COM, combined year; TRL, total root length; SA, surface area; LTL, lateral total length; NT, number of tips.

In this mapping population, root traits related to 39 QTLs were identified across 10 different chr. (3, 5, 6, 7, 8, 14, 15, 16, 17, and 18), including 17 co-located QTLs (Table 4 and Figure 3). The distribution of QTLs related to TRL, SA, LTL, and NT was observed across the 20 chr. of soybean mapping population (Figure S2).

A total of 10 TRL QTLs were identified on chr. 6, 7, 8, 14, 15, 16, and 17. Each QTL contributed to phenotypic variation (*R*^2^) ranging from 5.12% to 17.58% and had a logarithm of odds (LOD) score from 3.01 to 6.23. Among these QTLs, *qTRL-2022-14-1* exhibited the highest LOD value (6.23) and *R*^2^ (17.58%), with a marker position at 136.56 cM on chr. 14, where the favorable allele originated from the wild soybean “PI483463”. Eleven QTLs associated with SA were identified on chr. 5, 7, 8, 14, 15, 16, 17, and 18, with LOD scores ranging from 3.11 to 6.66. The *R*^2^ attributed to each QTL ranged from 5.52% to 19.92%. The *qSA-2022-14-1* was located on chr. 14 at a marker position of 136.56 cM, explaining an *R*^2^ of 19.92% with a LOD value of 6.66. The favorable allele was contributed by the wild soybean “PI483463” and cultivated soybean “Hutcheson” (Table 4 and Figure 3).

In total, 10 QTLs for LTL were identified across seven chr. 3, 5, 7, 8, 14, 15, and 16. The *R*^2^ and LOD scores associated with these QTLs varied, with percentages ranging from 6.40% to 13.39% and LOD scores from 3.34 to 5.44. The QTL, *qLTL-COM-15-1*, was located on chr. 15 at 9.66 cM, accounting for 13.39% of the *R*^2^ and a LOD score of 5.44; the positive allele provided from “PI483463”. There were eight NT QTLs across chr. 5, 8, 14, 15, 16, 17, and 18, explaining phenotypic variance ranging from 6.34% to 14.36%. Among these, *qNT-COM-5-1* exhibited the highest LOD value of 5.59 on chr. 5 at a location of 58.70 cM, with the favorable allele contributed by cultivated and wild soybean (Table 4 and Figure 3).

### 2.3. Root Trait QTLs with Positive Alleles from Wild Soybean

We identified the five most significant common QTL regions on five different chr., such as chr. 8, 14, 15, 16, and 17, which were associated with the root traits of TRL, SA, LTL, and NT where positive alleles were provided from wild soybean ‘PI483463’. Wild soybeans generally have a small root system, but the wild soybean accession “PI483463” was identified to carry beneficial alleles. These alleles have the potential to enhance overall root system architecture, allowing for targeted exploration of these QTL regions to identify candidate genes. The significant QTLs regions were observed within the following marker intervals: (Gm8_2547323_A_G~Gm8_2671408_T_C), (Gm14_7387315_T_G~Gm14_7778233_G_A), (Gm15_11927735_T_C~Gm15_12611331_A_G), (Gm16_3541782_T_C~Gm16_36809255_A_C), and (Gm17_33637862_T_C~Gm17_34217947_T_C) (Table 4, Figure 3). Additionally, some co-located QTLs for TRL, SA, LTL, and NT were observed on chr. 5, 8, and chr. 18, positioned at 58.70 cM, 8.46 cM, and 180.94 cM, respectively. The advantageous alleles for these root traits originated from wild soybean “PI483463” (Table 4 and Figure 3).

### 2.4. Putative Candidate Gene and Variant Analysis in Joint QTL Regions

Our results showed putative genes within the five most significant QTL regions on chr. 8, 14, 15, 16, and 17, respectively, which were common to different traits. There are 234 putative genes in the interval regions of the SNPs that are significantly associated with TRL, SA, LTL, and NT (Table S2). SNP variants were identified by comparing the parental sequences “Hutcheson” and “PI483463” with the reference genome sequences of “William 82” (https://soykb.org/SNPViz2/, accessed on 11 May 2024). Within the five potential QTL regions, 61 SNP variants were annotated as missense and splice region mutations (Table 5). These missense variants and splice region variations play a crucial role in amino acid alterations [30]. Additionally, Supplementary Table S3 provides detailed descriptions of the variants in genes, and these genes were responsible for amino acid alterations.

### 2.5. Gene Expression and Candidate Gene Identification

**Figure 4:**
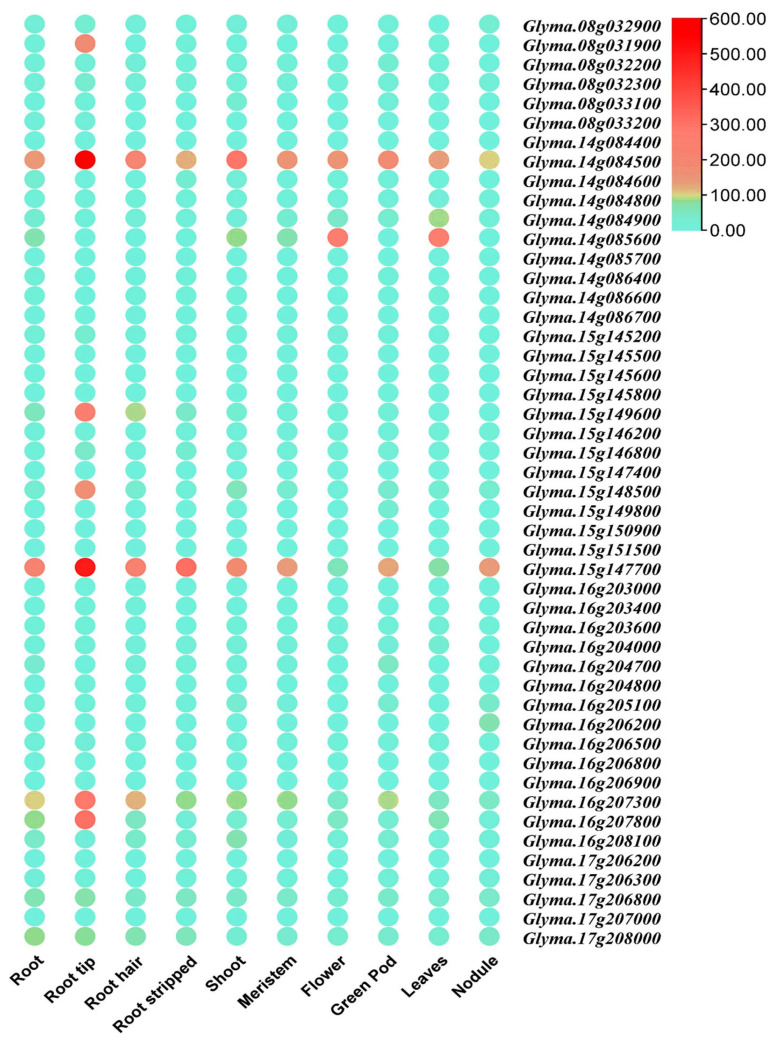
Expression profiles of tissue-specific data. A graduated color scale from light blue to red denotes the transcript levels, where red indicates higher expression while light blue shows less expression. Gene expression data were retrieved from ePlant Soybean Expression (https://bar.utoronto.ca/eplant_soybean/, accessed on 17 May 2024).

Tissue-specific transcriptome data such as root, root tip, root stripped, shoot, meristem, flower, green pod, leaves, and root nodule were obtained from the ePlant Soybean Expression (https://bar.utoronto.ca/eplant_soybean/, accessed on 17 May 2024) database to assess the expression of candidate genes (Table S4). Figure 4 illustrates the expression analysis result of candidate genes utilizing transcriptome data. Among the candidate genes, eight of them, *Glyma.16g205100* (Leucine-Rich Repeat Protein Kinase Family Protein), *Glyma.08g031900* (137-fold; NAC Domain Protein 75), *Glyma.14g084500* (145-fold; Polyadenylate-Binding Protein 2), *Glyma.15g149600* (55-fold; Drought Induced 21), *Glyma.15g148500* (252-fold; ATP Binding Cassette Transporter), *Glyma.15g147700* (217-fold; 40S Ribosomal Protein S21), *Glyma.16g207300* (167-fold; 30S/40S Ribosomal Protein S3), and *Glyma.16g207800* (1483-fold; Catalytic LigB Dioxygenase) were selected based on their locations in the QTL regions that were significantly associated with TRL, SA, LTL, and NT, as well as their higher expression in root tissues. The information was obtained from the SoyBase transcriptome database (http://www.soybase.org, accessed on 5 May 2024) and the ePlant Soybean Expression site (https://bar.utoronto.ca/eplant_soybean/, accessed on 17 May 2024) (Table 6, Figure 4).

## 3. Discussion

### 3.1. Phenotypic Variation in Root Morphological Traits

This study commenced with genotyping an interspecific mapping population using the GoldenGate^®^ assay, which initially includes 1536 SNP loci. This population consisted of 188 F_4:5_ RILs derived from the cross “Hutcheson” × “PI483463” [26,59]. From this initial dataset, we constructed a genetic map using 551 polymorphic molecular markers, including 535 SNP markers, after filtering. This map was used to identify QTL regions associated with salt tolerance in wild soybeans [60], soybean seed weight [26], forage yield and quality traits [61,62], and fresh shoot weight of soybeans [63]. To enhance homozygosity, we randomly selected a single plant from 185 F_10_ RILs and genotyped it using the BARCSoySNP3K SNP array. This array included a subset of 2680 SNP loci distributed across the 20 soybean chr., derived from the larger BARCSoySNP6K dataset [64]. Approximately 1200 polymorphic SNP markers were used to construct an improved genetic map, significantly refining the QTL positions for the linkage mapping study within this interspecific population.

Root morphological traits, such as TRL, SA, LTL, and NT, are essential for soybean production, including growth, development, and yield [22,65,66]. In this study, we employed a crossbreeding approach between the soybean varieties “Hutcheson” and “PI483463” to develop a population of RILs for mapping QTLs associated with root morphological characteristics. The main goal is to identify QTLs that regulate key root traits such as TRL, SA, LTL, and NT to facilitate future improvement in soybean root characteristics. Analysis of the parental lines and RILs population during the mapping process revealed significant variation in root attributes. The cultivated soybean “Hutcheson” exhibited a more vigorous root system than the wild soybean “PI483463” (Figure 1). The genetic recombination of chromosomal regions in the offspring of “Hutcheson” and “PI483463” exhibited a superior phenotypic value that exceeded that of the better parent, “Hutcheson” (Table 2). The differences observed in root architecture traits highlight the potential for targeted breeding efforts to enhance root system features, leading to improved resource uptake and increased resilience to stress. The same phenomenon occurs in interspecific mapping populations for root characteristics, leading to the discovery of new alleles that could enhance root morphological traits of soybeans [3,15,20,67].

The significance of root traits such as TRL and SA play a vital role in optimizing soil nutrient uptake, a key determinant of crop yield. Given the importance of TRL and SA in resource acquisition, developing specific root characteristics through breeding provides a valuable strategy to enhance crop productivity, particularly in regions with restricted arable land and under changing climate conditions [68]. Root morphological traits, particularly TRL and SA, are essential for enhancing plant productivity under climate change conditions such as drought [4]. Effective and extensive TRL and SA significantly enhanced crop resilience and yield potential [21,69,70]. This research highlights the significant differences in root traits, such as TRL, SA, and other root characteristics among different soybean accessions. These traits correlate with improved root hair development, drought resistance, mineral absorption, increased lateral root growth, and overall root development. In addition, studies on soybean root morphology show variations in TRL and SA in field environments. In this study, significant variations we identified in root characteristics among soybean accessions, including TRL, root SA, lateral TRL, and the number of root tips, observed within the same setting and across different years. The findings suggest a significant genetic diversity in the root morphological trait of soybeans, which may be leveraged to breed varieties that exhibit enhanced performance in various environmental conditions.

The correlation analysis of soybeans, as reported by Prince et al. [15], reveals multiple essential root traits, including TRL and SA, which significantly influence the variations observed in soybean root morphology and growth. These root morphological traits fundamentally influence how soybean plants interact with their environment, particularly in nutrient and water absorption, which are essential for growth and productivity. For instance, larger TRL showed a highly positive correlation with SA (*r* = 0.97), LTL (*r* = 0.77), and NT (*r* = 0.91). Thus, our findings suggest that TRL is influenced by SA, LTL, and NT. This correlation implies that these characteristics are interconnected and may collectively contribute to enhanced soil resource acquisition efficiency in plants. Similar results are observed for soybean [15,20], rice [71], sorghum [72], and chickpea [73], suggesting that TRL strongly associates with other root morphological traits, leading to improved root growth through enhanced water and nutrient uptake.

To date, studies on soybeans show some QTLs associated with root traits [1,3,14,15,20,27,74,75]. However, research focusing on mapping QTLs specifically for TRL, SA, LTL, and NT in soybeans is limited [1,15,20,75]. TRL-related QTLs were identified on chromosomes 5, 6, 8, 10, 11, 16, 18, and 20, explaining 7% to 30% of phenotypic variance in an interspecific soybean mapping population, while SA-related QTLs were located on chromosomes 7, 8, 10, 16, and 20, accounting for 6% to 12% of the phenotypic variance [15,20,67,75]. LTL-related QTLs were identified on chromosomes 3, 5, 8, 11, and 18, accounting for 8% to 13% of phenotypic variance, while NT-related QTLs were found on chromosomes 1, 9, 11, 13, and 20, exhibiting a broad range of phenotypic variance [75,76,77]. In this study, TRL was associated with six QTLs, SA with five QTLs, LTL with two QTLs, and NT with four QTLs, all of which were mapped on chromosomes 8, 14, 15, 16, and 17, with phenotypic variation ranging from 5.25% to 19.92%. Our findings indicate that certain genomic regions are present on different chr., such as chr. 14 and 15 for TRL, SA, and LTL, chr. 8, 14, 15, and 16 for NT root traits. Thus, this study shows novel QTL regions and favorable alleles provided from both cultivated and wild soybean accessions.

Our study identified SNPs associated with TRL, SA, LTL, and NT, revealing five significant QTL regions within the following marker intervals: (Gm8_2547323_A_G to Gm8_2671408_T_C), (Gm14_7387315_T_G to Gm14_7778233_G_A), (Gm15_11927735_T_C to Gm15_12611331_A_G), (Gm16_3541782_T_C to Gm16_36809255_A_C), and (Gm17_33637862_T_C to Gm17_34217947_T_C). The wild soybean accession, PI 483463, was found to possess beneficial alleles that have the potential to improve the overall root morphological traits. These alleles enable a targeted investigation of the associated QTL regions to identify candidate genes. Prince et al. [15] reported SNPs associated with TRL from NCSB_000550 to SNP5617_Magellan and from BARC_020495_04641 to BARC_023101_03769 and SNPs related to SA on SNP02285 to SNP18129_Magellan, with the beneficial alleles originating from the wild soybean PI438460B, which improved the root traits of soybean. Some researchers have indicated that root length enhances root development and stress resistance in soybeans [3,20,78] and rice [79,80].

### 3.2. Candidate Genes Underlying QTLs

This study shows SNP markers linked to root traits, such as TRL, SA, LTL, and NT, in significant QTL regions across five different chr., i.e., chr. 8, 14, 15, 16, and 17. We identified several missense and splice region SNP variations responsible for amino acid changes in soybeans [30]. QTLs are crucial in determining root traits by influencing various aspects of root development and function. In this study, we examined the association of SNPs with four root traits across diverse soybean accessions. Five significant SNP regions were identified for key root traits, including TRL, SA, LTL, and NT, located on chr. 8, 14, 15, 16, and 17. These QTL regions are vital for soybean genetic improvement, as they regulate root traits. Finally, we identified several genes within the specific genomic loci that directly or indirectly influence root function, potentially enhancing root growth and stress response in soybeans. The genes *Glyma.16g205100* and *Glyma.16g193600*, associated with TRL, SA, and NT on chr. 16, encode Leucine-rich repeat receptor protein kinases (LRR-RPKs) that regulate various growth, development, and physiological processes in plants [51]. *Glyma.16g193600* is identified as an abiotic stress-responsive gene that is upregulated among chitin-responsive differentially expressed genes (DEGs) and has functional links to *GmDR1* (a defense-related gene) [52]. Additionally, certain LRR-RKs are specifically expressed in the roots and nodules of *Medicago truncatula*, where they play a role in root meristem development [53,81]. In *Arabidopsis*, the *RGF1* INSENSITIVE 1–5 group in LRR-RKs plays a crucial role in detecting root meristem growth factor 1 [82]. The PSY1 peptide, another LRR-RK, enhances root length by promoting cell expansion and is ubiquitously expressed in the plant, including the shoot apical meristem and elongation zone of the root meristem [54]. RGF peptides, similar to GOLVEN and CLE-like peptides, regulate root development and the stem cell niche. Their bioactivity requires modifications, such as tyrosine sulfation [83,84]. They influence root meristem development via transcription factors (TFs) *PLETHORA1 (PLT1*) and *PLT2* [83]. LRR-RK genes are more significantly upregulated in roots than in stems and leaves because roots serve as the initial barrier to cadmium stress in *Sedum alfredii* [85].

The gene *Glyma.16g207800*, corresponding to *AT4G15093*, is associated with TRL and SA root traits and annotated as an iron-containing dioxygenase [86]. This gene, also known as *AtLigB*, encodes an extradiol ring-cleavage dioxygenase, a key enzyme in the biosynthesis of arabidopyrones (AP), commonly referred to as α-pyrone in other plant species [56]. α -pyrone is a secondary metabolite involved in plant defense, functioning as an antimicrobial agent and playing a crucial role in enhancing resistance to pathogens. α -pyrone plays a role in regulating seedling blight induced by *Fusarium moniliforme* and causing defense responses in maize [87]. It also controls the growth and defense responses in *Arabidopsis* [88]. In contrast, *AtLigB* homologs are widely conserved among field crops and many bacterial species, highlighting its evolutionary Fe^+2^ (GO:0008198) and zinc ion binding (GO:0008270), [57,58,89]. The gene *Glyma.08g031900*, located on chr. 8, is associated with root traits such as TRL, SA, and NT and encodes a NAC (No Apical Meristem, NAM) domain-containing protein 75. The NAC TF superfamily, a plant-specific group, encompasses a wide range of functions that are essential for plant growth, development, and stress adaptation. Research demonstrates that NAC TFs are pivotal in various developmental processes, including lateral root formation, maintenance of the shoot apical meristem, secondary cell wall biosynthesis, phytohormone signaling, and leaf senescence [31]. *Glyma.08g031900*, an ortholog of *AT4G29230*, encodes the NAC TF NAC075, specifically expressed in the root vascular cylinder [32]. This gene serves as a potential regulator of secondary cell wall development in *Arabidopsis* [90] by promoting the ectopic differentiation of xylem vessel elements, which leads to secondary cell wall deposition [91]. It plays a significant role in both salt and drought stress by interacting with TFs WRKY, bZIP, and ABA signaling (ABA1 and ABF3) in various crops [35,92,93,94]. It also responds to low nitrogen stress in sesame [95]. Some paralogs of NAC075 are reported in soybeans in response to high salinity, cold, dehydration, and ABA treatment [96,97,98]. This suggests that NAC075 influences secondary cell wall formation and plays a broader role in developmental timing and stress responses.

The gene *Glyma.15g149600*, located on chr. 15, is associated with TRL, SA, LTL, and NT and encodes the Drought-induced 21 (Di21) TF. *Glyma.15g149600* is expressed throughout the entire soybean plant, including roots, lateral roots, and root tips, showing enhanced gene expression in response to salt treatment. Overexpressing this gene in *Arabidopsis* confers increased drought tolerance [41]. Its homolog, *At4g15910*, encodes a protein that is upregulated under drought conditions and responds to ABA treatment and water deprivation, highlighting its role in stress responses [42]. *At4g15910* displays elevated expression levels in both 5-day-old and 17-day-old roots, as well as in callus, root cultures, and root tips [42]. Additionally, the ortholog gene (GO:0006950) regulates the root transcriptome in rice and maize under water and osmotic stress conditions [34,43]. The genes *Glyma.16g207300* and *Glyma.15g147700*, associated with root morphological traits (TRL, SA, NT) on chr. 15 and 16, encode the 40S Ribosomal Proteins S3 (RPS3) and S21 (RPS21), respectively. RPS3 undergoes important posttranslational modifications, including methylation, phosphorylation, and N-glycosylation [48]. In rice transgenic plants with ectopic *AtTOR* expression, both RPS3 and RPS21 are highly upregulated, resulting in increased root length and improved root activation [49]. Additionally, differentially expressed RPS21 in *Vitis riparia* × *V. labrusca* demonstrates enhanced root growth under cold stress [50].

The gene *Glyma.15g148500*, associated with TRL and SA root traits, encodes an ATP binding cassette (ABC) transporter that transports IAA, an auxin precursor, in plants [44,45,99]. The homolog in *Arabidopsis*, *AtABCB4*, exhibits concentration-dependent influx/efflux transporter activity [44]. Loss-of-function mutants of *AtABCB4* exhibit abnormal gravitropism, defective lateral root formation, and increased root hair elongation [44,100]. *AtABCB4*, along with *AtABCB1* and *AtABCB19*, influences polar auxin transport, a process critical for directional plant growth. Mutations in these ABCB transporters disrupt basipetal auxin transport in roots [44,101]. Unlike the polar localization of *PIN1* and *PIN2*, which drive auxin transport, ABCB proteins (ABCB1, ABCB4, and ABCB19) primarily display nonpolar and stable localization in the plasma membrane of root apexes [101,102]. ABCB proteins help to maintain the auxin gradient established by *PINs*, stabilize *PINs*, and, in some cases, function as polar auxin transporters [101,102]. ABCB proteins play diverse and complex roles in polar auxin transport. The gene *Glyma.14g084500* encodes poly(A) binding protein 2 (PABP2) and is associated with root traits of TRL, SA, LTL, and NT. *Glyma.14g084500* is predominantly expressed in root tips, root meristems, and lateral root primordia of seedlings and juvenile plants [37]. PABP2 enhances salt tolerance in *Arabidopsis*, *Glycine soja*, and *Glycine max* through the upregulation of key stress-responsive genes, such as *GmABI1*, *GmABI2*, *GmbZIP1*, *GmP5CS*, *GmCAT4*, *GmPIP1:6*, *GmMYB84*, and *GmSOS1* [38,39]. Furthermore, PABP2 also promotes root elongation in *Arabidopsis*, highlighting its significance in root development and stress adaptation [40]. Based on the preceding discussion, we hypothesize that key genes regulate soybean root growth and development and stress responses through direct and indirect mechanisms. This study shows markers and candidate genes that offer essential genetic resources for soybeans. Finally, our findings suggest that key root QTLs offer breeders opportunities to enhance soybean root morphological traits such as TRL, SA, LTL, and NT.

## 4. Materials and Methods

### 4.1. Plant Materials and Growth Conditions

In this investigation, we utilized a mapping population consisting of 185 F_10_ Recombinant Inbred Lines (RILs). This RIL population originated from a cross between the cultivated soybean variety “Hutcheson” and wild soybean accession “PI483463” [26]. The RILs and parental lines were grown in polyvinyl chloride (PVC) pipes, each measuring 6 cm in diameter and 40 cm in height, within a greenhouse at Kyungpook National University, Daegu, South Korea. The experiment was conducted over 2 years (22 August 2022 and 28 March 2023). In both experiments, two seeds from each parent and RILs were sown in the PVC pipes. The study utilized a completely randomized design with four replications. Sandy soil served as the growth medium for the experiment. In the greenhouse, a long day condition was applied to the soybean plants, providing 14 h of light and 10 h of darkness. After seed germination, thinning was performed to retain a single seedling per PVC pipe for subsequent root analysis. By day 29, most (>80%) of the seedlings reached the V3 (third trifoliate leaf) stage in both environments, and root samples were collected for analysis (Figure S3).

### 4.2. Evaluation of Root Morphological Traits

We used V3 seedlings for root image collection. After harvesting, we carefully removed all sandy soil from the PVC pipes and separated the root samples from the soil. These root samples were gently washed with clean tap water and then placed in plastic bags (20 cm long × 15 cm wide) with a small amount (15–20 mL) of water to retain moisture. A scanner (Epson, Expression 12,000XL, Nagano, Japan) was utilized to capture clear 2D root images. For the scanning process, a transparent plastic tray (30 cm long × 20 cm wide) filled with clean water was employed. The acquired root images were analyzed using WinRHIZO Pro software version 2019 (Regent Instruments Inc., Québec, Canada). QTL mappings were performed for the total root length (TRL; cm), surface area (SAcm^2^), lateral total length (LTL; cm), and number of tips (NT; count) for QTL mapping. The morphological traits of roots, as described in the study, were assessed using the WinRHIZO software, version 2019 (Table S5).

### 4.3. Construction of Linkage Map

F_10_ RILs (185) and their parental lines (“Hutcheson” and “PI483463”) were randomly selected for analysis [26,62]. Genomic DNA extracted from leaf samples was genotyped using the BARCSoySNP3K SNP array [103], which includes a subset of 2680 SNP loci distributed across 20 soybean chromosomes (chr.) from the BARCSoySNP6K array [64]. These SNP markers, developed by the soybean genomics and improvement laboratory at the USDA, were filtered to yield 1188 polymorphic markers between the parental lines (Table S2). We employed these polymorphic markers to develop a genetic linkage map for the 20 chr. IciMapping 4.2 software was used to construct the linkage map, employing the Kosambi mapping function to calculate the map distances between SNP markers [104].

### 4.4. QTL Analysis

In our study, WinQTLCart 2.5 was used for QTL analysis based on a composite interval mapping algorithm [105]. Model 6 was utilized with a window size of 10 cM and background cofactors. To identify significant QTLs, a permutation test (*p* = 0.05) was conducted with 1000 runs for all traits, using a more stringent LOD threshold. The forward regression approach, with a step size of 2 cM, was employed for QTL detection. The analysis integrated mean values for four distinct soybean root morphological traits. QTL map positions on the linkage maps were visualized using the MapChart 2.2 software [106].

### 4.5. Putative Candidate Genes, Variants, and Expression Prediction

We utilized the five most significant joint or co-located QTL regions on chr. 8, 14, 15, 16, and 17 to identify potential candidate genes, leveraging annotation from Soybase (http://www.soybase.org, accessed on 8 May 2024) and Phytozome (https://phytozome-next.jgi.doe.gov, accessed on 10 May 2024). The annotation was based on the “Wm82.a2.v1” whole genome sequence assembly. We identified putative genes within each interval flanked by significant SNPs (Dataset S1) using the genome browser. Subsequently, we extracted SNP and INDEL variants in the genomes of “Hutcheson” and “PI483463” using the Soykb (https://soykb.org/SNPViz2/, accessed on 11 May 2024) database with the “Wm82.a2.v1” reference genome. From the ePlant Soybean Expression (https://bar.utoronto.ca/eplant_soybean/, accessed on 17 May 2024) database, we downloaded expression values for putative candidate genes related to root and other traits. We then compared these expression values using a heatmap. Furthermore, we constructed a heatmap using data from the root and other tissues such as whole root, root tip, root stripped, shoot, meristem, flower, green pod, leaves, and root nodule, with the Tbtools (https://github.com/CJ-Chen/Tbtools, accessed on 24 June 2024) software.

### 4.6. Statistical Analysis

The root phenotypic data were sorted using Excel 2016 software (Microsoft, Redmond, WA, USA). Descriptive statistical analyses of trait frequency distribution were performed using IBM SPSS statistics 25 and analysis of variance (ANOVA) and correlation (Pearson correlation coefficients) analysis were performed using SAS 9.4 software (SAS Institute, Gary, NC, USA). Broad sense heritability (H^2^) of root traits was calculated using ANOVA results incorporating genotype effects.

$$H^{2}=\frac{V_{g}}{V_{g}+\frac{V_{ge}}{n}+\frac{V_{\varepsilon }}{rn}}$$

where *V_g_* indicates the genotype variance, *V_ge_* represents the variance of genotype and environment interaction, *V_ε_* denotes the residual error variance, *r* indicates the number of replications, and n refers to the number of environments.

## 5. Conclusions

Our study used QTL mapping to investigate root traits in soybean using interspecific crosses of “Hutcheson” and “PI483463”. We identified 39 QTL regions distributed across 10 chromosomes including 17 common QTL regions associated with different root traits, among which five QTL regions on chr. 8, 14, 15, 16 and 17 regulate critical root traits TRL, SA, LTL and NT. The desired alleles originate from the wild soybean “PI483463” and the cultivated soybean “Hutcheson”. These identified QTL regions have great potential for enhancing soybean root morphological traits. We identified several candidate genes in these important QTL regions that affect root morphological traits. These identified genes and associated SNP variants hold significant potential for future research, including gene expression analysis to elucidate their functional roles and validation of these SNPs as molecular markers. Such markers could be effectively utilized in breeding programs to enhance soybean root architecture through advanced approaches such as genetic transformation, genome editing, and marker-assisted selection. Overall, our results suggest that these important regions can help breeders improve soybean root morphological traits as well as stress responses by taking advantage of novel alleles from wild soybean “PI483463”.

## Figures and Tables

**Table 1:** Descriptive statistics of four root morphological traits in the soybean mapping population.

Traits	Parents		RIL Population					
	Hutcheson	PI483463	Minimum	Maximum	Mean	Range	Skewness	Kurtosis
**2022**								
TRL	1144.03	220.27	153.65	2166.03	934.46	2012.38	0.52	0.01
SA	94.93	27.69	17.02	228.61	98.93	211.59	0.57	0.3
LTL	144.98	77.61	32.68	244.53	149.37	211.85	0.23	0.03
NT	1023.5	218.33	170	2492	1019.55	2322	0.71	0.44
**2023**								
TRL	1043.78	255.03	246.91	1849.63	861.87	1602.72	0.35	−0.19
SA	87.31	27.7	25.42	219.12	107.51	193.7	0.28	−0.27
LTL	135.74	105.19	56.3	249.17	157.1	192.87	0.11	−0.2
NT	903	162.33	162.33	1841.5	780.7	1679.17	0.6	0.32
**2022 and 2023 Combined**								
TRL	1093.91	237.65	237.65	1791.69	898.16	1554.04	0.44	0.12
SA	91.12	27.7	22.81	201.42	103.22	178.61	0.37	−0.04
LTL	140.36	91.4	68.25	233.62	153.23	165.37	0.14	−0.1
NT	963.25	190.33	190.33	1987.42	900.12	1797.09	0.69	0.66

**Note:** TRL, total root length; SA, surface area; LTL, lateral total length; NT, number of tips.

**Table 2:** Analysis of variance (F-value) for the four root traits in the interspecific mapping population of soybeans.

Source	df	TRL	SA	LTL	NT
Genotype	184	146.58 ***	303.70 ***	1.78 ***	40.75 ***
Environment	1	368.36 ***	853.29 ***	3.26 ^ns^	1043.11 ***
Replication	3	0.995 ^ns^	1.37 ^ns^	0.91 ^ns^	0.91 ^ns^
Genotype × Environment	184	61.08 ***	97.30 ***	1.66 ***	17.52 ***
H^2^		70.8%	75.7%	68.2%	70.0%

**Note:** *** indicates the level of significance at *p* < 0.0001; ns indicate no significant difference. ×—interaction. df, degree of freedom; TRL, total root length; SA, surface area; LTL, lateral total length; and NT, number of tips; H^2^, heredity.

**Table 3:** Pearson correlation analysis for the four root traits in the soybean mapping population.

	TRL	SA	LTL	NT
TRL	1	0.97 ***	0.77 ***	0.91 ***
SA	0.96 ***	1	0.75 ***	0.85 ***
LTL	0.72 ***	0.73 ***	1	0.72 ***
NT	0.91 ***	0.86 ***	0.67 ***	1

**Note:** *** indicates the level of significance at *p* < 0.0001. TRL, total root length; SA, surface area; LTL, lateral total length; and NT, number of tips. Black color data (upper right corner) represents the year 2022, while the blue color (lower left matrix) data indicates the year 2023.

**Table 4:** Detected root trait QTLs on different chromosomes of the soybean mapping population using composite interval mapping (CIM).

Trait	QTL Name	Chr.	Left Marker	Right Marker	Position (cM)	LOD	*R*^2^ (%)	Additive
LTL-2022	qLTL-2022-3-1	3	03_39915523_C_T	03_40052612_T_C	154.83	4.48	10.69	−12.99
NT-COM	qNT-COM-5-1	5	05_33843473_C_T	05_34294649_G_T	58.70	5.59	14.36	109.60
LTL-COM	qLTL-COM-5-1	5	05_33843473_C_T	05_34294649_G_T	58.70	5.17	13.30	9.81
NT-2023	qNT-2023-5-1	5	05_33843473_C_T	05_34294649_G_T	58.70	3.49	6.49	1.87
SA-COM	qSA-COM-5-1	5	05_32327497_T_C	05_3284858_A_G	45.74	3.11	5.52	0.44
SA-2022	qSA-2022-6-1	6	06_16390152_A_G	06_17399306_A_C	32.37	3.79	7.18	11.06
SA-COM	qSA-COM-7-1	7	07_2267894_G_A	07_2704982_G_A	41.46	4.05	8.50	−9.81
LTL-COM	qLTL-COM-7-1	7	07_8488086_A_G	07_8887938_C_T	127.08	3.34	6.55	−7.77
TRL-COM	qTRL-COM-7-1	7	07_43946876_A_G	07_4562451_T_C	99.80	3.07	5.12	87.04
SA-2023	qSA-2023-8-1	8	08_2547323_A_G	08_2671408_T_C	87.27	5.18	12.73	−12.31
TRL-COM	qTRL-COM-8-1	8	08_13640846_A_G	08_14646649_A_G	27.96	4.65	10.77	92.40
LTL-2023	qLTL-2023-8-1	8	08_10621107_C_A	08_10947242_C_T	6.46	4.21	9.05	−9.86
TRL-2023	qTRL-2023-8-1	8	08_2547323_A_G	08_2671408_T_C	87.27	3.66	7.01	−84.62
LTL-COM	qLTL-COM-8-1	8	08_10621107_C_A	08_13109130_A_G	7.46	3.42	6.40	−8.18
NT-2023	qNT-2023-8-1	8	08_2547323_A_G	08_2671408_T_C	87.27	3.25	6.53	−78.61
SA-COM	qSA-COM-8-1	8	08_10621107_C_A	08_13109130_A_G	8.46	3.17	6.38	9.32
SA-2022	qSA-2022-14-1	14	14_7387315_T_G	14_7778233_G_A	136.56	6.66	19.92	−17.39
TRL-2022	qTRL-2022-14-1	14	14_7387315_T_G	14_7778233_G_A	136.56	6.23	17.58	−170.18
LTL-2022	qLTL-2022-14-1	14	14_7387315_T_G	14_7778233_G_A	134.34	5.10	12.06	−14.29
NT-COM	qNT-COM-14-1	14	14_7387315_T_G	14_7778233_G_A	136.56	3.36	6.32	−100.07
LTL-COM	qLTL-COM-15-1	15	15_11927735_T_C	15_1209819_T_C	9.66	5.44	13.39	−11.76
LTL-COM	qLTL-COM-15-2	15	15_11192460_G_A	15_11496274_T_C	2.64	4.95	11.78	−9.77
SA-COM	qSA-COM-15-1	15	15_12140462_T_G	15_12611331_A_G	20.35	4.72	10.83	10.75
TRL-COM	qTRL-COM-15-1	15	15_12140462_T_G	15_12611331_A_G	20.66	4.15	8.90	88.42
LTL-2023	qLTL-2023-15-1	15	15_2391075_G_A	15_2729636_A_C	42.93	4.03	7.38	9.80
NT-2022	qNT-2022-15-1	15	15_12140462_T_G	15_12611331_A_G	20.66	3.15	6.34	120.09
TRL-2023	qTRL-2023-15-1	15	15_45725175_G_A	15_46014592_T_C	85.36	3.12	6.16	79.04
SA-2022	qSA-2022-16-1	16	16_36413821_C_A	16_36809255_A_C	121.46	5.92	14.49	−14.17
TRL-2022	qTRL-2022-16-1	16	16_36413821_C_A	16_36809255_A_C	121.46	4.91	11.52	−127.04
LTL-2022	qLTL-2022-16-1	16	16_2800650_C_T	16_28232079_A_G	88.73	4.06	8.77	−14.42
NT-COM	qNT-COM-16-1	16	16_35322776_G_A	16_35700223_G_T	117.00	3.91	7.57	90.85
TRL-COM	qTRL-COM-16-1	16	16_35322776_G_A	16_35700223_G_T	116.14	3.01	5.25	89.83
SA-2022	qSA-2022-17-1	17	17_33637862_T_C	17_34217947_T_C	45.49	4.75	10.92	−12.86
SA-2022	qSA-2022-17-2	17	17_20787666_A_G	17_22702978_G_T	36.16	4.42	9.35	−12.49
TRL-2022	qTRL-2022-17-1	17	17_33637862_T_C	17_34217947_T_C	45.49	3.61	6.59	−109.42
NT-2023	qNT-2023-17-1	17	17_6576837_G_T	17_6778459_T_G	108.67	3.56	6.64	82.83
TRL-COM	qTRL-COM-17-1	17	17_1736595_A_G	17_17971540_A_G	34.80	3.15	6.35	80.00
NT-2023	qNT-2023-18-1	18	18_8937974_T_C	18_9382031_T_C	180.94	3.77	7.26	86.07
SA-2023	qSA-2023-18-1	18	18_8937974_T_C	18_9382031_T_C	180.94	3.25	6.41	−9.83

**Note:** Chr., chromosome; COM, combined year; LOD, logarithm of the odds; *R*^2^, percentage of phenotypic variation explained; TRL, total root length; SA, surface area; LTL, lateral total length; NT, number of tips. In additive effect, positive value indicates the favorable alleles provided from “Hutcheson”, and (−) indicates favorable alleles contributed from “PI483463”.

**Table 5:** SNP variants with genes underlying the significant joint QTL regions of the soybean mapping population.

SNP Position	Gene Name	Hut-Cheson	PI483463	Ref.	Mutation Type	Start Physical Position of the Gene (bp)	End Physical Position of the Gene (bp)	Strand
Chr08:2550484	*Glyma.08g031900*	G	T	T	Missense variant	2,547,941	2,553,643	+
Chr08:2576236	*Glyma.08g032200*	A	T	T	Missense variant	2,575,678	2,578,818	−
Chr08:2589180	*Glyma.08g032300*	G	C	C	Missense variant	2,587,418	2,590,621	+
Chr08:2621158	*Glyma.08g032900*	G	T	G	Missense variant	2,618,659	2,623,500	+
Chr08:2634722	*Glyma.08g033100*	G	T	T	Missense variant	2,634,444	2,637,284	−
Chr08:2650616	*Glyma.08g033200*	A	G	G	Missense variant	2,649,700	2,651,523	−
Chr14:7401505	*Glyma.14g084400*	T	G	T	Missense variant	7,400,306	7,405,768	+
Chr14:7401758	*Glyma.14g084400*	A	T	A	Missense variant	7,400,306	7,405,768	+
Chr14:7402135	*Glyma.14g084400*	G	C	G	Missense variant	7,400,306	7,405,768	+
Chr14:7409761	*Glyma.14g084500*	C	T	C	Splice region variant	7,409,216	7,414,343	+
Chr14:7426690	*Glyma.14g084600*	A	C	A	Splice region variant	7,425,379	7,427,280	−
Chr14:7478433	*Glyma.14g084800*	C	T	C	Missense variant	7,473,827	7,479,302	+
Chr14:7481634	*Glyma.14g084900*	C	G	C	Missense variant	7,480,720	7,481,721	−
Chr14:7587937	*Glyma.14g085600*	C	A	C	Missense variant	7,587,535	7,588,825	+
Chr14:7596878	*Glyma.14g085700*	G	A	G	Missense variant	7,596,635	7,597,069	+
Chr14:7730748	*Glyma.14g086400*	G	A	A	Missense variant	7,728,210	7,731,282	−
Chr14:7747716	*Glyma.14g086600*	A	T	T	Missense variant	7,747,225	7,748,170	−
Chr14:7747723	*Glyma.14g086600*	C	G	G	Missense variant	7,747,225	7,748,170	−
Chr14:7754046	*Glyma.14g086700*	T	G	G	Splice region variant	7,753,865	7,755,101	−
Chr15:11951066	*Glyma.15g145200*	A	G	G	Splice region variant	11,947,682	11,957,050	−
Chr15:11984075	*Glyma.15g145500*	T	C	T	Missense variant	11,983,796	11,985,371	−
Chr15:11994025	*Glyma.15g145600*	T	C	T	Missense variant	11,992,826	11,994,293	+
Chr15:12015158	*Glyma.15g145800*	A	C	A	Missense variant	12,015,121	12,015,979	+
Chr15:12039437	*Glyma.15g146200*	A	C	A	Splice region variant	12,035,374	12,039,865	−
Chr15:12076661	*Glyma.15g146800*	T	A	T	Missense variant	12,076,031	12,079,129	−
Chr15:12154491	*Glyma.15g147400*	T	C	T	Missense variant	12,152,905	12,154,821	+
Chr15:12218870	*Glyma.15g148500*	C	G	G	Missense variant	12,216,661	12,226,663	+
Chr15:12372053	*Glyma.15g149600*	C	T	C	Missense variant	12,371,767	12,375,710	−
Chr15:12487011	*Glyma.15g150900*	T	C	T	Missense variant	12,477,309	12,487,462	−
Chr15:12557945	*Glyma.15g151500*	A	G	A	Missense variant	12,557,700	12,567,175	−
Chr17:33655483	*Glyma.17g206200*	G	T	G	Missense variant	33,655,181	33,655,853	+
Chr17:33655511	*Glyma.17g206200*	T	G	T	Missense variant	33,655,181	33,655,853	+
Chr17:33737053	*Glyma.17g206300*	G	C	G	Missense variant	33,730,503	33,738,647	−
Chr17:33737055	*Glyma.17g206300*	A	T	A	Missense variant	33,730,503	33,738,647	−
Chr17:33885152	*Glyma.17g206800*	A	G	A	Missense + Splice region variant	33,884,210	33,886,354	−
Chr17:33930371	*Glyma.17g207000*	A	G	A	Missense variant	33,925,621	33,935,113	+
Chr17:34058444	*Glyma.17g208000*	T	G	T	Missense variant	34,093,823	34,096,844	−
Chr17:34108428	*Glyma.17g208100*	C	T	C	Missense variant	34,108,328	34,108,849	−
Chr17:34108738	*Glyma.17g208100*	T	C	T	Missense variant	34,108,328	34,108,849	−
Chr16:35333627	*Glyma.16g190900*	C	G	C	Missense variant	35,333,029	35,336,258	+
Chr16:35374182	*Glyma.16g191300*	T	A	A	Missense variant	35,372,401	35,375,711	+
Chr16:35545878	*Glyma.16g193200*	T	C	C	Missense variant	35,545,733	35,547,841	+
Chr16:35550031	*Glyma.16g193600*	G	C	G	Missense variant	35,576,270	35,580,207	+
Chr16:35663355	*Glyma.16g194400*	G	A	G	Missense variant	35,659,212	35,664,619	+
Chr16:36412977	*Glyma.16g203000*	T	G	G	Missense variant	36,412,774	36,413,868	−
Chr16:36412983	*Glyma.16g203000*	A	G	G	Missense variant	36,412,774	36,413,868	−
Chr16:36413223	*Glyma.16g203000*	G	A	A	Missense variant	36,412,774	36,413,868	−
Chr16:36451102	*Glyma.16g203400*	C	A	C	Missense variant	36,449,021	36,451,535	+
Chr16:36457878	*Glyma.16g203600*	A	T	A	Missense variant	36,457,598	36,461,359	+
Chr16:36511200	*Glyma.16g204000*	A	G	G	Missense variant	36,507,514	36,511,554	−
Chr16:36570923	*Glyma.16g204700*	A	G	A	Missense variant	36,570,193	36,572,473	+
Chr16:36583397	*Glyma.16g204800*	C	T	C	Missense variant	36,583,127	36,586,048	+
Chr16:36607850	*Glyma.16g205100*	G	A	G	Missense variant	36,607,332	36,611,337	−
Chr16:36664087	*Glyma.16g206200*	T	A	A	Missense variant	36,663,932	36,664,834	−
Chr16:36678618	*Glyma.16g206500*	G	A	A	Missense variant	36,675,871	36,679,332	+
Chr16:36684470	*Glyma.16g206800*	G	T	T	Missense variant	36,684,395	36,684,947	-
Chr16:36688416	*Glyma.16g206900*	C	T	C	Missense variant	36,686,943	36,688,802	-
Chr16:36719823	*Glyma.16g207300*	G	T	G	Splice region variant	36,718,287	36,720,700	+
Chr16:36751621	*Glyma.16g207800*	A	T	T	Missense variant	36,750,028	36,751,833	−
Chr16:36777525	*Glyma.16g208100*	T	G	G	Missense variant	36,776,806	36,778,169	+

**Note:** Ref, reference allele from “William 82” (Wm82.a2.v1 reference genome assembly). (+) sign indicates the sense strand of DNA, while the (−) sign denotes the antisense strand of DNA.

**Table 6:** Final candidate genes, directly and indirectly, affect significant root morphological traits.

Gene	Start Physical Position (bp)	End Physical Position (bp)	Gene Annotation	Gene Roles	References
*Glyma.08g031900*	2547941	2553643	NAC Domain Containing Protein 75 (NAC075)	promotes lateral root growth, root elongation, shoot apical meristem, ABA regulation, and abiotic stress response.	[31,32,33,34,35,36]
*Glyma.14g084500*	7409216	7414343	Polyadenylate-Binding Protein 2 (PABP2)	increase root length, root hairs, flower development, and response to salt stress.	[37,38,39,40]
*Glyma.15g149600*	12350959	12352153	Drought Induced 21 (Di21)	plays a vital role in root growth by increasing root tip and root meristem, induced ABA, and drought and salt stress response	[41,42,43]
*Glyma.15g148500*	12216661	12226663	ATP-Binding Cassette (ABC) Transporter	regulates root growth by modulating auxin and cytokinin levels and helps in adaptation and defense response.	[44,45,46,47]
*Glyma.15g147700*	12165892	12167660	40S Ribosomal Protein S21 (RPS21)	controls primary root and shoot development, ensures stronger root activity against cold resistance, and enhances drought and salt resistance	[48,49,50]
*Glyma.16g205100*	36607332	36611337	Leucine-Rich Repeat Receptor Protein Kinase Family Protein (LRR-RPKs)	regulates auxin transport, root, nodule, root meristem development, and response in salt, drought, cold, and heat stresses.	[51,52,53,54]
*Glyma.16g207300*	36718287	36720700	40S Ribosomal Protein S3 (RPS3)	helps in auxin signaling, narrow leaf development, lateral and crown root formation, and cold and drought stress response.	[48,49,50,55]
*Glyma.16g207800*	36750028	36751833	Catalytic LigB Dioxygenase	regulates iron, zinc uptake, α-pyrone, and isoflavonoid biosynthesis.	[56,57,58]

## Data Availability

Data contained within the article and Supplementary Materials.

## Supplementary Materials

The following supporting information can be downloaded at https://www.mdpi.com/article/10.3390/ijms26031152/s1.
