# Supplementary material for: Integration of Genetic and Imaging Data to Detect QTL for Root Traits in Interspecific Soybean Populations
Source: Int J Mol Sci. 2025 Jan 28;26(3):1152. doi: 10.3390/ijms26031152 (PMC11817972; doi:10.3390/ijms26031152)
Supplement: Supplementary file 1 [file ijms-26-01152-s001.zip › ijms-3417779-supplementary.pdf]

Integration of Genetic and Imaging Data to Detect QTL for Root Traits in Interspecific Soybean Populations

Table S1. Map information and distribution of polymorphic markers across the 20 chromosomes.

Chr.	Linkage Group	No. Marker	Length (cM)	Distance/Marker
1	D1a	52	123.7	2.38
2	D1b	56	174.4	3.11
3	N	63	219.2	3.48
4	C1	53	160.5	3.03
5	A1	47	136.8	2.91
6	C2	53	192	3.62
7	M	62	142	2.29
8	A2	80	219	2.74
9	K	52	155	2.98
10	O	58	161	2.78
11	B1	47	126.2	2.69
12	H	36	153.4	4.26
13	F	67	248.3	3.71
14	B2	54	148.6	2.75
15	E	82	157.8	1.92
16	J	49	161.4	3.29
17	D2	65	126.2	1.94
18	G	78	181	2.32
19	L	73	134.6	1.84
20	I	61	135.7	2.22
	Total	1188	3256.8	2.74

Table S2. List of total 234 putative genes in the significant SNP marker regions on chromosome 8, 14, 15, 16, and 17 for TRL, SA, LTL, and NT root traits.

Putative genes in the significant region (**Gm08:2547323..2671408**) on chromosome 8			
Glyma_08g032900	*Glyma_08g031900*	*Glyma_08g032000*	*Glyma_08g032100*
Glyma_08g032200	*Glyma_08g032300*	*Glyma_08g032400*	*Glyma_08g032500*
Glyma_08g032600	*Glyma_08g032700*	*Glyma_08g032800*	*Glyma_08g033000*
Glyma_08g033100	*Glyma_08g033200*		
Putative genes in the significant region (**Gm14:7387315..7778233**) on chromosome 14			
Glyma_14g084300	*Glyma_14g084400*	*Glyma_14g084500*	*Glyma_14g084600*
Glyma_14g084700	*Glyma_14g084800*	*Glyma_14g084900*	*Glyma_14g085000*
Glyma_14g085100	*Glyma_14g085200*	*Glyma_14g085300*	*Glyma_14g085400*
Glyma_14g085500	*Glyma_14g085600*	*Glyma_14g085700*	*Glyma_14g085800*
Glyma_14g085900	*Glyma_14g086000*	*Glyma_14g086100*	*Glyma_14g086200*
Glyma_14g086300	*Glyma_14g086400*	*Glyma_14g086500*	*Glyma_14g086600*
Glyma_14g086700	*Glyma_14g086800*	*Glyma_14g086900*	
Putative genes in the significant region (**Gm15:11927735..12611331**) on chromosome 15			
Glyma_15g151900	*Glyma_15g146400*	*Glyma_15g148100*	*Glyma_15g150000*
Glyma_15g148200	*Glyma_15g146500*	*Glyma_15g148300*	*Glyma_15g150100*
Glyma_15g149300	*Glyma_15g146600*	*Glyma_15g148400*	*Glyma_15g150200*
Glyma_15g145000	*Glyma_15g146700*	*Glyma_15g148500*	*Glyma_15g150300*
Glyma_15g145100	*Glyma_15g146800*	*Glyma_15g148600*	*Glyma_15g150400*
Glyma_15g145200	*Glyma_15g146900*	*Glyma_15g148700*	*Glyma_15g150500*
Glyma_15g145300	*Glyma_15g147000*	*Glyma_15g148800*	*Glyma_15g150600*
Glyma_15g145400	*Glyma_15g147100*	*Glyma_15g148900*	*Glyma_15g150700*
Glyma_15g145500	*Glyma_15g147200*	*Glyma_15g149000*	*Glyma_15g150800*
Glyma_15g145600	*Glyma_15g147300*	*Glyma_15g149100*	*Glyma_15g150900*
Glyma_15g145700	*Glyma_15g147400*	*Glyma_15g149200*	*Glyma_15g151000*
Glyma_15g145800	*Glyma_15g147500*	*Glyma_15g149400*	*Glyma_15g151100*
Glyma_15g145900	*Glyma_15g147600*	*Glyma_15g149500*	*Glyma_15g151200*
Glyma_15g146000	*Glyma_15g147700*	*Glyma_15g149600*	*Glyma_15g151300*
Glyma_15g146100	*Glyma_15g147800*	*Glyma_15g149700*	*Glyma_15g151400*
Glyma_15g146200	*Glyma_15g147900*	*Glyma_15g149800*	*Glyma_15g151500*

Glyma_15g146300	*Glyma_15g148000*	*Glyma_15g149900*	*Glyma_15g151600*
Glyma_15g151700	*Glyma_15g151800*	*Glyma_15g152000*	
Putative genes in the significant region (**Gm16:35322776..36809255**) on chromosome 16			
Glyma_16g190800	*Glyma_16g193300*	*Glyma_16g203500*	*Glyma_16g206000*
Glyma_16g190900	*Glyma_16g193400*	*Glyma_16g203600*	*Glyma_16g206100*
Glyma_16g191000	*Glyma_16g193500*	*Glyma_16g203700*	*Glyma_16g206200*
Glyma_16g191100	*Glyma_16g193600*	*Glyma_16g203800*	*Glyma_16g206300*
Glyma_16g191200	*Glyma_16g193700*	*Glyma_16g203900*	*Glyma_16g206400*
Glyma_16g191300	*Glyma_16g193800*	*Glyma_16g204000*	*Glyma_16g206500*
Glyma_16g191400	*Glyma_16g193900*	*Glyma_16g204100*	*Glyma_16g206600*
Glyma_16g191500	*Glyma_16g194000*	*Glyma_16g204200*	*Glyma_16g206700*
Glyma_16g191600	*Glyma_16g194100*	*Glyma_16g204300*	*Glyma_16g206800*
Glyma_16g191700	*Glyma_16g194200*	*Glyma_16g204400*	*Glyma_16g206900*
Glyma_16g191800	*Glyma_16g194300*	*Glyma_16g204500*	*Glyma_16g207000*
Glyma_16g191900	*Glyma_16g194400*	*Glyma_16g204600*	*Glyma_16g207100*
Glyma_16g192000	*Glyma_16g194500*	*Glyma_16g204700*	*Glyma_16g207200*
Glyma_16g192100	*Glyma_16g194600*	*Glyma_16g204800*	*Glyma_16g207300*
Glyma_16g192200	*Glyma_16g194700*	*Glyma_16g204900*	*Glyma_16g207400*
Glyma_16g192300	*Glyma_16g194800*	*Glyma_16g205000*	*Glyma_16g207500*
Glyma_16g192400	*Glyma_16g194900*	*Glyma_16g205100*	*Glyma_16g207600*
Glyma_16g192500	*Glyma_16g195000*	*Glyma_16g205200*	*Glyma_16g207700*
Glyma_16g192600	*Glyma_16g195100*	*Glyma_16g205300*	*Glyma_16g207800*
Glyma_16g192700	*Glyma_16g195200*	*Glyma_16g205400*	*Glyma_16g207900*
Glyma_16g192800	*Glyma_16g203300*	*Glyma_16g205500*	*Glyma_16g208000*
Glyma_16g192900	*Glyma_16g203000*	*Glyma_16g205600*	*Glyma_16g208100*
Glyma_16g193000	*Glyma_16g203100*	*Glyma_16g205700*	*Glyma_16g208200*
Glyma_16g193100	*Glyma_16g203200*	*Glyma_16g205800*	*Glyma_16g208300*
Glyma_16g193200	*Glyma_16g203400*	*Glyma_16g205900*	*Glyma_16g208400*
Putative genes in the significant region (**Gm17:33637862..34217947)** on chromosome 17			
Glyma_17g206200	*Glyma_17g206700*	*Glyma_17g207200*	*Glyma_17g207700*
Glyma_17g206300	*Glyma_17g206800*	*Glyma_17g207300*	*Glyma_17g207800*
Glyma_17g206400	*Glyma_17g206900*	*Glyma_17g207400*	*Glyma_17g207900*

Glyma_17g206500	*Glyma_17g207000*	*Glyma_17g207500*	*Glyma_17g208000*
Glyma_17g206600	*Glyma_17g207100*	*Glyma_17g207600*	*Glyma_17g208100*
Glyma_17g208200	*Glyma_17g208300*		

Table S3. Identified putative candidate gene with function annotation description on the significant QTLs regions.

Gene Name	**Gene Annotation Descriptions**
Glyma.08g032900	HEAT SHOCK PROTEIN 90
Glyma.08g031900	NAC DOMAIN CONTAINING PROTEIN 75/NO APICAL MERISTEM (NAM) PROTEIN
Glyma.08g032200	PROTEIN OF UNKNOWN FUNCTION (DUF1005)
Glyma.08g032300	DER1-LIKE FAMILY
Glyma.08g033100	NEUROMODULIN
Glyma.08g033200	MATE EFFLUX FAMILY PROTEIN
Glyma.14g084400	NUCLEAR FRAGILE X MENTAL RETARDATION-INTERACTING PROTEIN 1 (NUFIP1)
Glyma.14g084500	POLYADENYLATE-BINDING PROTEIN (RRM SUPERFAMILY)
Glyma.14g084600	HOMEOBOX DOMAIN
Glyma.14g084800	UNCHARACTERIZED MEMBRANE PROTEIN, PREDICTED EFFLUX PUMP
Glyma.14g084900	ZINC FINGER PROTEIN
Glyma.14g085600	CYSTEINE PROTEASE FAMILY C1-RELATED
Glyma.14g085700	CALCIUM-BINDING EF-HAND FAMILY PROTEIN
Glyma.14g086400	DOMAIN OF UNKNOWN FUNCTION (DUF303)
Glyma.14g086600	F-BOX DOMAIN
Glyma.14g086700	MEMBRANE PROTEIN
Glyma.15g145200	RESPONSE REGULATOR OF TWO-COMPONENT SYSTEM
Glyma.15g145500	PROTEIN OF UNKNOWN FUNCTION (DUF789)
Glyma.15g145600	PATHOGENESIS-RELATED PROTEIN BET V I FAMILY
Glyma.15g145800	DEFENSE RESPONSE
Glyma.15g149600	DROUGHT-INDUCED 21
Glyma.15g146200	MELANOMA-ASSOCIATED ANTIGEN (MAGE ANTIGEN)
Glyma.15g146800	GLYCOSYL HYDROLASES FAMILY 28
Glyma.15g147400	COPPER TRANSPORT PROTEIN ATOX1-RELATED
Glyma.15g148500	ABC TRANSPORTER
Glyma.15g149800	RAG1-ACTIVATING PROTEIN 1
Glyma.15g150900	ACYL-COA DEHYDROGENASE
Glyma.15g151500	60S RIBOSOMAL PROTEIN L11-RELATED
Glyma.15g147700	40S RIBOSOMAL PROTEIN S21
Glyma.16g190900	LEUCINE-RICH REPEAT RECEPTOR-LIKE PROTEIN KINASE
Glyma.16g191300	LEUCINE-RICH REPEAT RECEPTOR-LIKE PROTEIN KINASE
Glyma.16g193200	CAP160 PROTEIN
Glyma.16g193600	LEUCINE-RICH REPEAT RECEPTOR-LIKE PROTEIN KINASE
Glyma.16g194400	METHYL-CPG BINDING DOMAIN
Glyma.16g203000	F-BOX FAMILY PROTEIN

Glyma.16g203400	HIT-TYPE ZINC FINGER FAMILY PROTEIN
Glyma.16g203600	AMINOTRANSFERASE CLASS I AND II
Glyma.16g204000	ATP-DEPENDENT CLP PROTEASE
Glyma.16g204700	NUCLEIC ACID BINDING
Glyma.16g204800	PPR REPEAT
Glyma.16g205100	LEUCINE-RICH REPEAT PROTEIN KINASE FAMILY PROTEIN
Glyma.16g206200	SMALL HEAT-SHOCK PROTEIN (HSP20) FAMILY
Glyma.16g206500	RIBOSOMAL PROTEIN L2
Glyma.16g206800	ZINC ION BINDING
Glyma.16g206900	CLATHRIN ASSEMBLY PROTEIN
Glyma.16g207300	30S/40S RIBOSOMAL PROTEIN S3
Glyma.16g207800	CATALYTIC LIGB SUBUNIT OF AROMATIC RING-OPENING DIOXYGENASE
Glyma.16g208100	MEMBER OF 'GDXG' FAMILY OF LIPOLYTIC ENZYMES
Glyma.17g206200	ZINC ION BINDING
Glyma.17g206300	ATP-BINDING CASSETTE TRANSPORTER
Glyma.17g206800	MITOCHONDRIAL RIBOSOMAL PROTEIN L24
Glyma.17g207000	CARBON CATABOLITE REPRESSOR PROTEIN 4
Glyma.17g208000	UBIQUINOL-CYTOCHROME-C REDUCTASE ACTIVITY
Glyma.17g208100	14-3-3 PROTEIN/PROTEIN DOMAIN SPECIFIC BINDING

Table S4. Tissue specific transcriptome expression value of putative candidate genes from ePlant Soybean Expression (https:// ePlant (utoronto.ca)/eplant_soybean/).

**Gene Name**	Root	Root tip	Root hair	Root stripped	Shoot	Meristem	Flower	Green Pod	Leaves	Nodule
Glyma.08g032900	18.20	24.72	13.88	7.51	14.44	14.44	4.15	10.25	2.49	24.47
Glyma.08g031900	4.48	181.55	0.28	3.11	0.76	1.55	1.78	0.68	2.84	0.53
Glyma.08g032200	6.07	12.36	17.22	5.70	9.12	6.07	5.34	21.18	2.84	0.27
Glyma.08g032300	16.35	29.36	14.44	14.25	11.91	11.91	6.23	11.62	7.10	12.77
Glyma.08g033100	0.53	4.02	2.22	1.55	24.82	1.19	1.19	12.98	0.71	0.27
Glyma.08g033200	12.66	13.29	2.50	1.55	1.27	0.30	0.30	0.00	0.00	0.00
Glyma.14g084400	0.00	0.00	0.00	0.00	0.00	0.00	0.00	0.00	0.00	0.00
Glyma.14g084500	145.58	557.62	209.64	119.71	294.59	156.85	156.85	180.39	139.92	103.99
Glyma.14g084600	24.26	0.00	0.28	19.95	15.45	2.67	2.67	6.83	2.49	0.27
Glyma.14g084800	4.22	9.89	3.89	2.59	9.88	8.88	7.71	8.88	17.76	3.19
Glyma.14g084900	21.10	8.34	14.99	10.62	17.22	21.10	44.48	29.38	90.91	10.37
Glyma.14g085600	66.72	0.00	0.83	1.81	85.62	66.72	267.45	0.00	271.67	0.80
Glyma.14g085700	0.00	0.00	0.00	0.00	0.00	0.00	0.00	0.00	0.00	0.00
Glyma.14g086400	12.92	0.62	2.22	3.89	2.03	1.78	1.78	4.10	1.42	0.27
Glyma.14g086600	5.01	0.93	7.50	2.59	6.33	2.05	1.78	2.05	4.26	1.06
Glyma.14g086700	0.00	0.00	0.00	0.00	0.00	0.00	0.00	0.00	0.00	0.00
Glyma.15g145200	11.08	25.34	9.16	8.81	10.13	10.13	13.94	8.20	8.52	1.06
Glyma.15g145500	0.00	0.00	0.00	0.00	0.00	0.00	0.00	0.00	0.00	0.00
Glyma.15g145600	0.26	0.00	0.56	0.00	0.00	0.00	0.00	0.00	0.00	0.80
Glyma.15g145800	0.00	0.00	0.00	0.00	0.00	0.00	0.00	0.00	0.00	0.00
Glyma.15g149600	55.12	252.17	93.57	40.94	16.72	6.15	2.97	6.15	1.78	0.27
Glyma.15g146200	7.38	5.25	1.94	7.25	9.63	5.32	4.15	8.20	3.55	5.32
Glyma.15g146800	9.49	39.87	9.44	20.73	14.44	9.49	5.34	15.72	3.55	0.00
Glyma.15g147400	0.53	0.00	0.56	0.26	1.01	0.00	0.00	0.00	0.00	0.00
Glyma.15g148500	24.26	170.59	28.32	12.96	55.98	30.05	8.60	32.11	22.02	30.05
Glyma.15g149800	1.05	0.00	0.00	0.00	4.05	1.05	0.30	22.55	2.13	0.27
Glyma.15g150900	0.00	0.00	0.00	0.00	0.00	0.00	0.00	0.00	0.00	0.00

Glyma.15g151500	0.00	0.00	0.00	0.00	0.00	0.00	0.00	0.00	0.00	0.00
Glyma.15g147700	217.31	498.47	229.36	310.41	183.90	139.89	60.49	131.19	74.58	139.89
Glyma.16g203000	0.00	0.00	0.00	0.00	0.00	0.00	0.00	0.00	0.00	0.00
Glyma.16g203400	1.85	4.64	3.05	1.55	0.76	0.89	0.89	0.00	0.36	1.86
Glyma.16g203600	3.43	4.02	1.67	2.07	4.56	4.56	7.71	4.78	11.72	3.72
Glyma.16g204000	10.02	9.89	6.66	13.21	12.41	10.38	10.38	17.08	21.31	2.39
Glyma.16g204700	34.02	1.55	11.66	5.44	11.65	6.91	0.59	49.20	1.78	6.91
Glyma.16g204800	1.05	2.78	0.83	0.78	1.27	1.05	0.59	0.68	1.78	0.00
Glyma.16g205100	12.13	8.96	6.66	6.22	28.88	12.13	1.48	29.38	2.49	32.98
Glyma.16g206200	0.00	0.00	0.00	0.26	0.00	0.00	0.00	0.00	0.00	65.69
Glyma.16g206500	10.02	17.61	10.55	6.74	8.87	7.10	3.26	5.47	7.10	4.79
Glyma.16g206800	0.00	0.00	0.00	0.00	0.00	0.00	0.00	0.00	0.00	0.00
Glyma.16g206900	0.79	0.00	0.83	0.00	0.51	0.00	0.00	0.00	0.71	0.00
Glyma.16g207300	103.64	288.51	119.12	82.14	85.87	85.87	37.66	93.61	51.14	43.62
Glyma.16g207800	83.34	307.11	51.09	17.10	24.82	25.28	43.29	25.28	60.02	10.37
Glyma.16g208100	38.50	17.00	33.32	26.17	67.13	17.00	15.72	25.96	13.85	10.64
Glyma.17g206200	0.00	0.00	0.00	0.00	0.00	0.00	0.00	0.00	0.00	0.00
Glyma.17g206300	17.41	9.89	1.67	6.22	6.84	9.89	1.78	10.93	3.55	15.96
Glyma.17g206800	59.34	69.22	41.93	51.56	44.83	40.16	23.72	33.48	29.83	40.16
Glyma.17g207000	0.00	0.00	0.00	0.00	0.00	0.00	0.00	0.00	0.00	0.00
Glyma.17g208000	84.13	76.64	64.70	61.67	20.52	30.75	29.65	30.75	29.83	42.29

Table S5. Description of root morphological traits used in the study by WinRHIZO software.

Traits	Description
Total root length (TRL)	The sum of the Euclidean distances between the connected skeletal pixels in the entire root topology of the plant root image.
Surface area (SA)	For each pixel of the skeleton image of the root the SA is calculated for every punctual diameter of the root at that particular pixel position and then summed up to give the total surface area.
Lateral total length (LTL)	The total length of links of order 1 (first lateral branch of the root).
Number of tips (NT)	The number of root tips is pixels in identified root topology that have only one neighboring skeletal pixel.

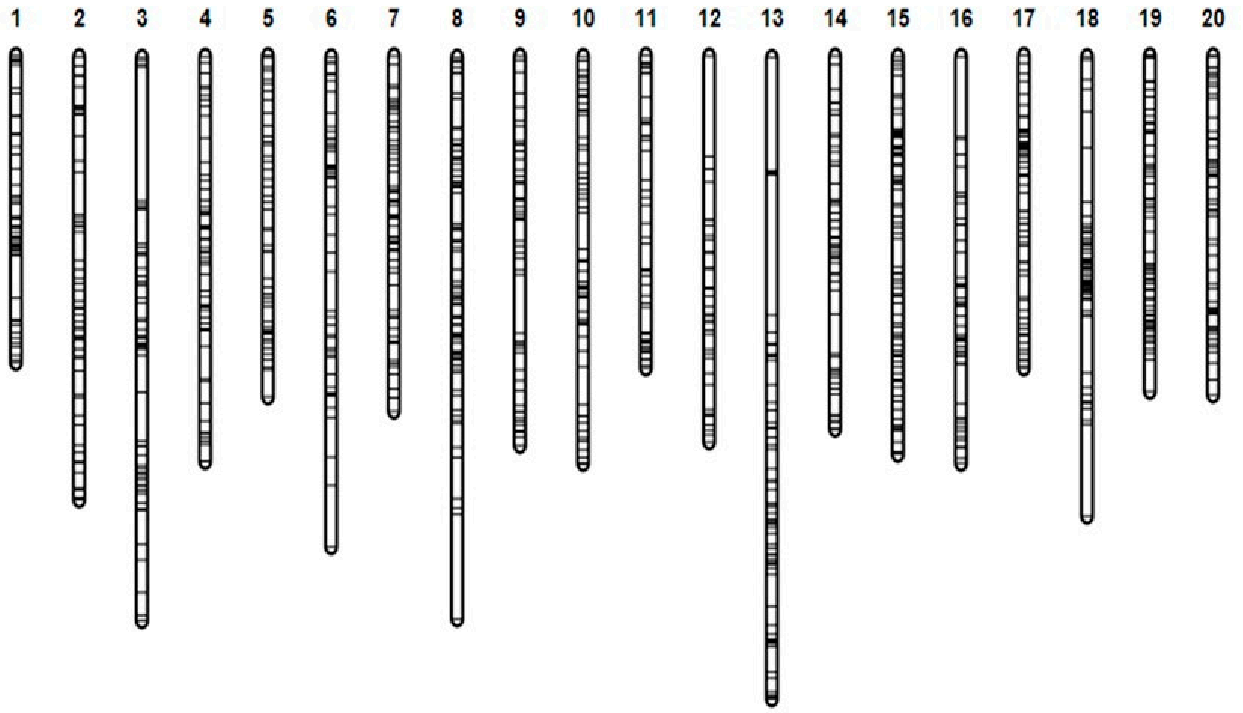

Figure S1. Distribution of SNP markers across the twenty (1-20) chromosomes in an inter-specific mapping population of soybean.

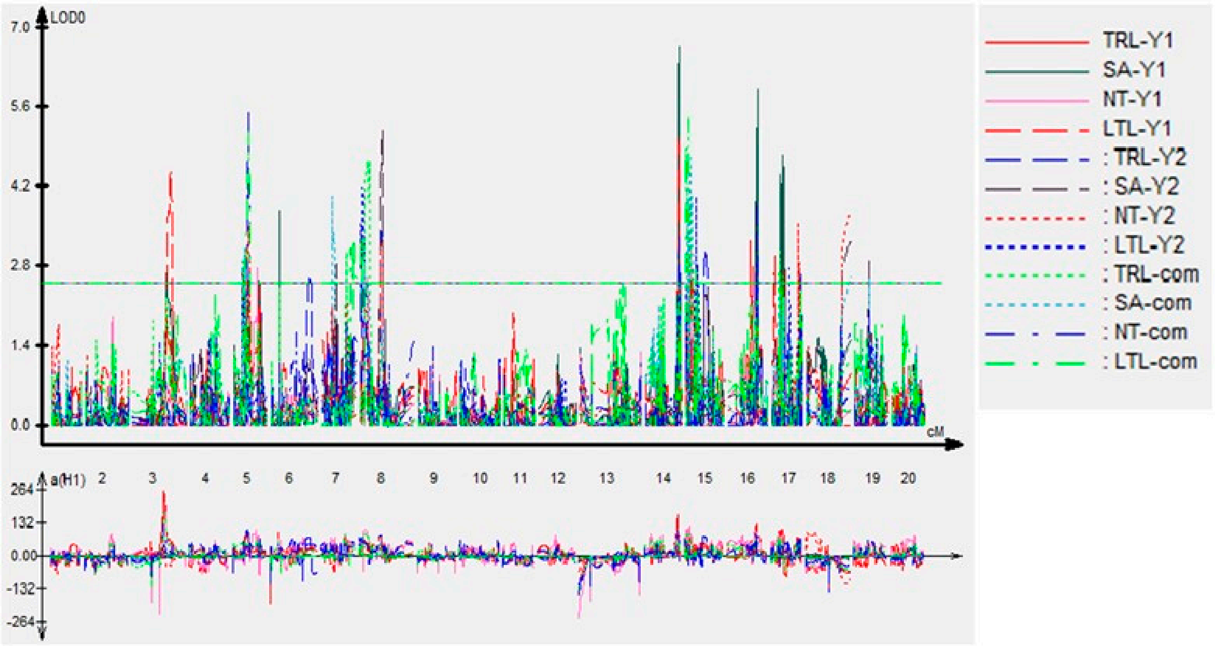

Figure S2. Diagram shown the distribution root traits TRL, SA, LTL, and NT QTLs on 20 different chromosomes. Different color indicates different root traits QTL. Y1, year 1; Y2, year 2; COM, combined year; TRL, total root length; SA, surface area; LTL, lateral total length; NT, number of tips.

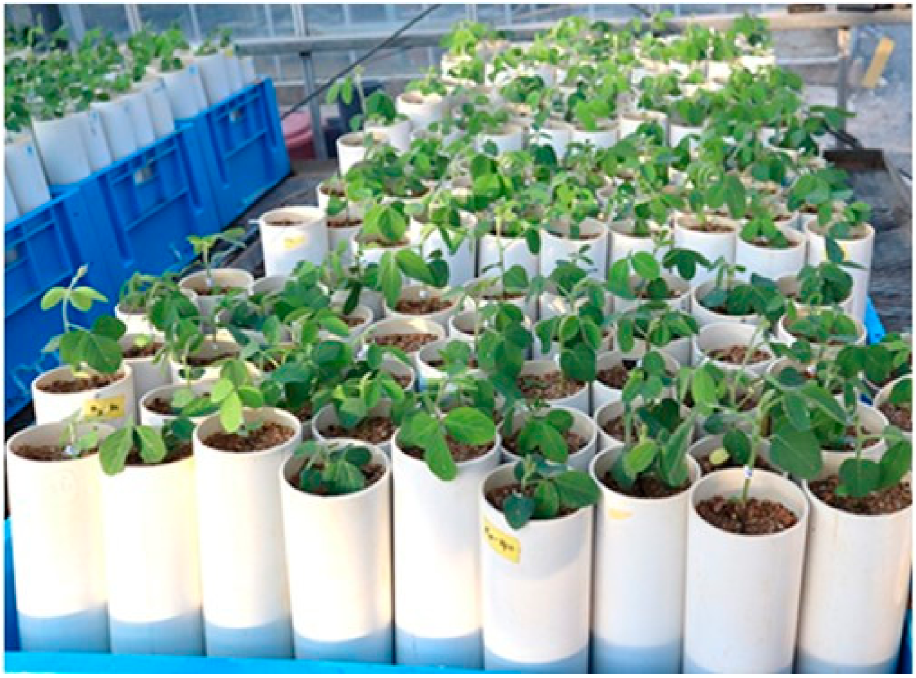

Figure S3. At 29 days aged soybean seedlings during harvesting period.
